# A novel microbe-drug association prediction model based on stacked autoencoder with multi-head attention mechanism

**DOI:** 10.1038/s41598-023-34438-8

**Published:** 2023-05-06

**Authors:** Liu Fan, Lei Wang, Xianyou Zhu

**Affiliations:** 1 College of Computer Science and Technology, Hengyang Normal University, Hengyang, 421010 China; 2 Institute of Bioinformatics Complex Network Big Data, Changsha University, Changsha, 410022 China; 3 Big Data Innovation and Entrepreneurship Education Center of Hunan Province, Changsha University, Changsha, 410022 China

**Keywords:** Computational biology and bioinformatics, Genetics, Mathematics and computing

## Abstract

Microbes are intimately tied to the occurrence of various diseases that cause serious hazards to human health, and play an essential role in drug discovery, clinical application, and drug quality control. In this manuscript, we put forward a novel prediction model named MDASAE based on a stacked autoencoder (SAE) with multi-head attention mechanism to infer potential microbe-drug associations. In MDASAE, we first constructed three kinds of microbe-related and drug-related similarity matrices based on known microbe-disease-drug associations respectively. And then, we fed two kinds of microbe-related and drug-related similarity matrices respectively into the SAE to learn node attribute features, and introduced a multi-head attention mechanism into the output layer of the SAE to enhance feature extraction. Thereafter, we further adopted the remaining microbe and drug similarity matrices to derive inter-node features by using the Restart Random Walk algorithm. After that, the node attribute features and inter-node features of microbes and drugs would be fused together to predict scores of possible associations between microbes and drugs. Finally, intensive comparison experiments and case studies based on different well-known public databases under 5-fold cross-validation and 10-fold cross-validation respectively, proved that MDASAE can effectively predict the potential microbe-drug associations.

## Introduction

Microbe colony are mainly composed of bacteria, viruses, etc.^1^. It normally survives on various human tissues, providing protection against pathogens, and can harmonize the homeostasis of the body's internal environment and regulate the pathology of the gastrointestinal tract to promote the body’s metabolic capacity^2,3^. Furthermore, ecological dysbiosis or imbalance of microbes may also lead to other diseases in the human host. For example, there are several pathways through which an imbalance of intestinal bacteria in the human body will lead to the risk of hypertension^4^. It is thus clear that the microbe is important to human health and many microbes presenting in the human organism can regulate host physiology and disease development^5,6^.

In recent years, as the variety of drugs investigated by the medical field increases, the resistance of microbes is becoming more and more intense^7^. Previous research in the pharmaceutical industry has involved culturing some microbe species under greenhouse conditions and subsequently using them in drugs^8^. However, this process is usually time- and money-consuming. This urgently requires researchers to adopt new computational methods to discover potential relationships between microbes and drugs, thus contributing to drug development assays and human disease diagnosis.

Until now, a great number of researchers have constructed a series of microbe and drug association databases in order to calculate potential links between microbes and drugs. For instance, Sun et al.^9^ established the MDAD, which is a database consisting of 5505 associations between 180 microbes and 1388 drugs. Rajput et al.^10^ concluded a database called aBiofilm, in which the resistance of microbes to drugs is recorded and biological, chemical, and structural details of 5027 antimicrobial film agents were contained as well. Moreover, Andersen et al. built a dataset called DrugVirus, which includes 1281 associations between 118 compound drugs and 83 human viruses^11^. Based on above databases, many computational models have been proposed successively to infer potential microbe-drug associations in the past few years. For example, Zhu et al. designed a computational method HMDAKATZ based on the KATZ measure to predict latent microbe-drug associations^12^. Long et al.^13^ proposed a method called EGATMDA to predict associations between microbes and drugs by using a graph convolutional network with node-level attention to learn embeddings of nodes and graph-level attention to learn the importance of different graphs. In 2020, Long et al. introduced a calculation method named GCNMDA based on the Graph Convolutional Network and Conditional Random Field with an attention mechanism to detect latent microbe-drug associations^14^. Deng et al.^15^ devised a method called Graph2MDA in 2021, which can predict potential associations of microbes with drugs by constructing a multimodal property graph as input to a variational graph autoencoder to learn information about each node and the whole graph. In 2022, Tan et al. constructed a model named GSAMDA based on a graph attention network and sparse autoencoder to compute microbe-drug correlations^16^. In 2023, Ma applied two heterogeneous microbe-drug networks as inputs of a graph attention network to learn feature representations of microbes and drugs, and then used a convolutional neural network classifier to obtain potential microbe-drug associations^17^. Predicting associations between biological entities is one of the fundamental tasks in the field of bioinformatics, and it is not only in microbe-drug association prediction that researchers have achieved excellent performance, there are also many splendid methods worth learning in areas like microbe-disease association prediction, circRNA-disease association prediction, predicting interactions between molecules and miRNA, and so on. For example, in 2022, Chen proposed a method called MATHNMDA^18^, which based on heterogeneous network and metapath aggregated graph neural network to predict microbe-disease associations. Peng et al. designed a model called GATCL2CD^19^, built a heterogeneous network by computing multiple similarities between circRNA and diseases, and proposed a feature convolution learning with heterogeneous graph attention network to predict circRNA-disease associations. In addition, Peng et al. used a deep autoencoder to obtain potential feature representations of each small molecule-miRNA pair as well as a scalable tree boosting model to predict potential associations with them^20^.

Despite the fact that above models have performed reliably in some aspects, there are certain limitations to them. With the rapid development of deep learning techniques in the last few years, numerous techniques become available to extract data features. In this paper, we present a new approach named MDASAE to infer potential microbe-drug associations based on a stacked autoencoder (SAE) with a multi-head attention mechanism. In MDASAE, we first adopt the restart random walk algorithm to learn inter-node features for microbes and drugs respectively based on the Gaussian kernel similarity. And then, we apply SAE with multi-head attention to extract node attribute features for microbes and drugs based on multiple similarity metrics in an unsupervised manner. Ultimately, we fuse these microbe- and drug-related features together to estimate association scores of different microbe-drug pairs. Besides, in order to evaluate the prediction performance of MDASAE, intensive comparison experiments are done based on two different well-known public databases, and experimental results show that MDASAE outperforms representative competitive methods, which means that it is practical and effective to apply multi-head attention mechanism to the stacked autoencoder for prediction of latent microbe-drug associations.

## Materials and methods

**Figure 1:**
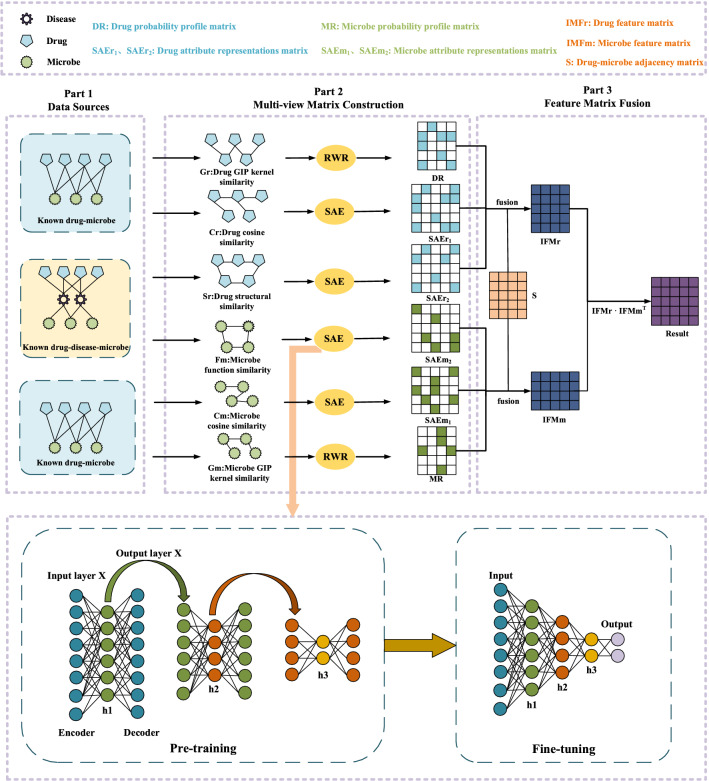
Flowchart of MDASAE.

As shown in Fig. 1. MDASAE is comprised of three main components. Among them, the first part is the preparation of data source including downloading known drug-microbe associations, microbe-disease associations, and drug-disease associations from well-known public databases. The second part is the construction of multi-view correlation matrices, including the microbe similarity matrix and the drug similarity matrix, based on different similarity measures, some of which will be used as inputs to a SAE with multi-head attention to learn node attribute features for microbes and drugs separately, while the remaining of which will be utilized to learn inter-node features for microbes and drugs respectively by adopting the RWR. In the third part, these newly-obtained node attribute features and inter-node features of microbes and drugs will be integrated together to obtain the final predicted scores of microbe-drug associations.

### Data sources

In this section, we download known microbe-drug associations from the MDAD (http://www.chengroup.cumt.edu.cn/MDAD/) first, which contains 5505 known microbe-drug associations between 1388 drugs and 180 microorganisms collected from 993 papers. Based on the de-duplication operations proposed by Wang et al.^21^, we finally obtain 2470 known microbe-drug associations between 173 microbes and 1373 drugs. And then, after removing duplicate microbe-drug associations, we further download 2884 known microbe-drug associations between 1720 drugs and 140 microbes from the aBiofilm (http://bioinfo.imtech.res.in/manojk/abiofilm/) for validation. The detailed data of the datasets download from above two well-known public databases are shown in Table 1. And furthermore, for convenience, we have kept all newly-downloaded datasets of diseases, drugs, microbes, drug-disease associations, drug-drug interactions, microbe-drug associations, microbe-disease associations and microbe-microbe interactions in Supplementary Information 1–8 separately.

**Table 1:** Statistics of two well-known public databases including MDAD and aBiofilm.

Datasets	Microbes	Drugs	Associations
MDAD	173	1373	2470
aBiofilm	140	1720	2884

### Methods

Based on newly-downloaded known microbe-drug association data from the datasets, let ${N}_{r}$ and ${N}_{m}$ denote the numbers of different drugs and microbes in the downloaded datasets, then it is obvious that we can build a microbe-drug adjacency matrix $S\in {R}^{{N}_{r}\times {N}_{m}}$ as follows: If and only if there is a known association between any given drug ${r}_{i}$ and microbe ${m}_{j}$, we define that there is $S(i,j)=1$, otherwise we define that there is $S(i,j)=0$.

#### Construction of the drug similarity network

Firstly, for any two given drugs ${r}_{i}$ and ${r}_{j}$, let $Rs(i)$ and $Rs(j)$ represent the *i*-th row and *j*-th row of *S* respectively, then we can calculate score of the Gaussian kernel similarity $Gr\left({r}_{i},{r}_{j}\right)$ between ${r}_{i}$ and ${r}_{j}$ is as follows:

1 $$ Gr\left( {r_{i} ,r_{j} } \right) = {\text{exp}}\left( { - \mu \Vert Rs\left( i \right) - Rs\left( j \right)\Vert^{2} } \right) $$

Here, $\mu$ denotes the standard nuclear bandwidth, which can be calculated as follows:

2 $$ \mu = 1/\left( {\frac{1}{{N_{r} }}\mathop \sum \limits_{i = 1}^{{N_{r} }} \Vert Rs\left( i \right)\Vert^{2} } \right) $$

Next, we can further obtain score of the drug Cosine similarity $Cr\left({r}_{i},{r}_{j}\right)$ between ${r}_{i}$ and ${r}_{j}$ is as follows:

3 $$ Cr\left( {r_{i} ,r_{j} } \right) = \frac{Rs\left( i \right) \cdot Rs\left( j \right)}{{\Vert Rs\left( i \right)\Vert \times \Vert Rs\left( j \right)\Vert }} $$

Finally, based on the chemical structural information existing between drugs, for any two given drugs ${r}_{i}$ and ${r}_{j}$, we will estimate score of structural similarity $Sr\left({r}_{i},{r}_{j}\right)$ between them by adopting the method of SIMCOMP2 proposed by Hattori et al.^22^.

#### Construction of the microbe similarity network

In a similar way, for any given microbes ${m}_{i}$ and ${m}_{j}$, let $Cs(i)$ and $Cs(j)$ represent the *i*-th column and *j*-th column of *S* separately, then we can first calculate score of the Gaussian kernel similarity $Gm\left({m}_{i},{m}_{j}\right)$ between ${m}_{i}$ and ${m}_{j}$ as follows:

4 $$Gm\left({m}_{i},{m}_{j}\right)=\mathrm{exp}(-\gamma {\Vert Cs(i)-Cs(j)\Vert }^{2})$$

5 $$ \gamma = 1/\left( {\frac{1}{{N_{m} }}\mathop \sum \limits_{i = 1}^{{N_{m} }} \Vert Cs\left( i \right)\Vert^{2} } \right) $$

And then, we can further calculate score of the Cosine similarity between ${m}_{i}$ and ${m}_{j}$ as follows:

6 $$Cm\left({m}_{i},{m}_{j}\right)=\frac{Cs(i)\cdot Cs(j)}{\Vert Cs(i)\Vert \times \Vert Cs(j)\Vert }$$

Moreover, we will further obtain score of the function similarity $Fm\left({m}_{i},{m}_{j}\right)$ between ${m}_{i}$ and ${m}_{j}$ based on the method proposed by Kamneva et al.^23^.

#### Feature extraction for drugs and microbes based on RWR

Due to the imbalance between positive and negative sample data, it may contain noise in the newly-obtained microbe and drug Gaussian kernel similarities. Inspired by the method of NTSHMDA^24^, in this section, we will further apply the algorithm of Restart Random Walk (RWR) to derive inter-node features for microbes and drugs respectively based on the Gaussian kernel similarity, since RWR has been effectively utilized in miRNA-disease association prediction^25^, lncRNA-disease association prediction^26^, and target gene identification^27^ respectively. Here, the RWR adopted in MDASAE is defined as follows^28^:

7 $${q}_{i}^{l+1}=\lambda M{q}_{i}^{l}+(1-\lambda ){e}_{i}$$

where $\lambda$ is the restart probability, which will be set to 0.1 in MDASAE, and *M* is the transition probability matrix. Besides, ${e}_{i}\in {R}^{(1\times m)}$ is the original probability vector of node *i* in the microbe or drug Gaussian kernel similarity network, which is defined as follows:

8 $$ e_{i,j} = \left\{ \begin{gathered} 1\quad if\   i = j \hfill \\ 0\quad otherwise \hfill \\ \end{gathered} \right. $$

Based on above methods, it is easy to see that we can obtain a drug probability profile matrix *DR* and a microbe probability profile matrix *MR* eventually.

#### Learning attribute features for microbes and drugs based on SAE

Stacked autoencoder (SAE) is composed of stacked layers with several Autoencoders (AEs) that mainly consist of three layers such as the input layer, the hidden layer, and the output layer. In order to learn the attribute features between nodes, SAE is first pre-trained in an unsupervised manner, and then a supervised method is employed to fine-tune the parameters in the SAE. To be specific, SAE could learn the most important attributes of the input data, and reconstruct the input data in the output layer through encoding and decoding. Generally, the stage of mapping the input data to the hidden layer through a nonlinear activation function is called encoding, and the mapping of the hidden layer to the output layer is called decoding. In this section, in order to extract the attribute features of drugs and microbes more efficiently, we will adopt $Cr$ and $Sr$ as the input of SAE respectively to learn the attribute features of drugs, and $Cm$ and $Fm$ as the input of SAE respectively to learn the attribute features of microbes.

For convenience, let *X* denote the input of SAE, then the output of the hidden layer in the encoding process of SAE can be defined as follows:

9 $$Y=\sigma (WX+b)$$

where $\sigma$, *W* and *b* represent the activation function, the weight matrix and the bias of the hidden layer in the encoding phase of SAE respectively.

And additionally, in the decoding process, the input *X* will be reconstructed according to the following formula:

10 $$ X^{\prime } = \sigma \left( {W^{\prime } Y + b^{\prime } } \right) $$

where $W^{\prime }$ and $b^{\prime }$ denote the weight matrix and bias of the hidden layer in the decoding phase of SAE separately.

Considering that the input *X* may not be fully reconstructed in the decoding phase, we further add a multi-head attention mechanism in SAE to capture critical features and improve the efficiency and accuracy of the attribute feature extraction process.

Finally, we will introduce the Adam optimizer for training in SAE, which is more efficient than the traditional SGD optimizer. The Adam optimizer is calculated as follows:

11 $$L\left(X,{X}^{^{\prime}}\right)={\Vert X-{X}^{^{\prime}}\Vert }^{2}={\Vert X-\sigma ({W}^{^{\prime}}\left(\sigma \left(WX+b\right)\right)+{b}^{^{\prime}})\Vert }^{2}$$

Based on above methods, it is obvious that, by adopting SAE, we can obtain two different drug attribute feature matrices and two different microbe attribute feature matrices respectively. For simplicity, we define these two drug attribute feature matrices as $SAE{r}_{1}$ and $SAE{r}_{2}$, and these two microbe attribute feature matrices as $SAE{m}_{1}$ and $SAE{m}_{2}$, separately.

#### Predicting association scores of microbe-drug pairs

Firstly, we stack the drug probability profile matrix *DR* and the two different drug attribute feature matrices $SAE{r}_{1}$ and $S{AEr}_{2}$ horizontally. Meanwhile, in order to fuse more raw data information about drugs and to ensure a matched dimension of the integrated drug feature matrix, we also add an adjacency matrix *S*. It is easy to see that we can obtain an integrated drug feature matrix $IFMr$ as follows:

12 $$IFMr=\left[DR;S;S{AEr}_{1};S;SAE{r}_{2};S\right]$$

And then, in a similar way, through combining the microbe probability profile matrix *MR*, the two different microbe attribute feature matrices $S{AEm}_{1}$ and $SAE{m}_{2}$ with the adjacency matrix *S*. we can obtain an integrated microbe feature matrix $IFMm$ as follows:

13 $$IFMm=\left[{S}^{T};MR;{S}^{T};S{AEm}_{1};{S}^{T};SAE{m}_{2}\right]$$

where $$IFMr\in {R}^{{N}_{r}\times (({N}_{r}+{N}_{m})\times 3)}$$ and $$IFMm\in {R}^{{N}_{m}\times (({N}_{m}+{N}_{r})\times 3)}$$.

Finally, in order to simulate the interaction of drugs and microbes, we can obtain the predicted scores of associations between them by adopting the inner product of $IFMr$ and $IFMm$. In general, the multiplication of two vectors is a means of simulating the interaction of two different objects, which on the one hand weakens the information about their differences and on the other hand highlights the commonality of the interactions. The formula is as follows:

14 $$Score=Sigmoid(IFMr\cdot {IFMm}^{T})$$

where ${IFMm}^{T}$ is the transposed matrix of $IFMm$ and *Sigmoid* is an activation function.

## Results

In this section, we will first analyze the effects of relevant parameters on the predictive performance of MDASAE for model optimization. And then, an ablation experiment will be employed to analyze the effect of the multi-head attention mechanism on the prediction performance MDASAE, followed by performance comparison between MDASAE and five state-of-the-art competitive prediction methods. Eventually, some specific drugs and microbes will be selected out as case studies to confirm the validity of MDASAE.

### Hyperparameter sensitivity analysis

From above descriptions, it is easy to see that there are several key parameters in MDASAE such as the learning rate *l*_*r*_ for model training, the number of attention mechanism heads *h* in SAE, as well as the neurons number *n*_1_ of the hidden layer and the number* n*_2_ of stacked AEs in SAE, and so on. In this section, we will employ the 5-fold cross-validation to evaluate the prediction performance MDASAE and adjust the values of parameters by observing the mean of AUCs achieved on MDAD. When implementing the 5-fold cross-validation, we will randomly divide those downloaded microbe-drug pairs into five equal subsets first, and then, each subset will be selected out in turn to serve as the test set, while the remaining four subsets serve as the training set.

**Figure 2:**
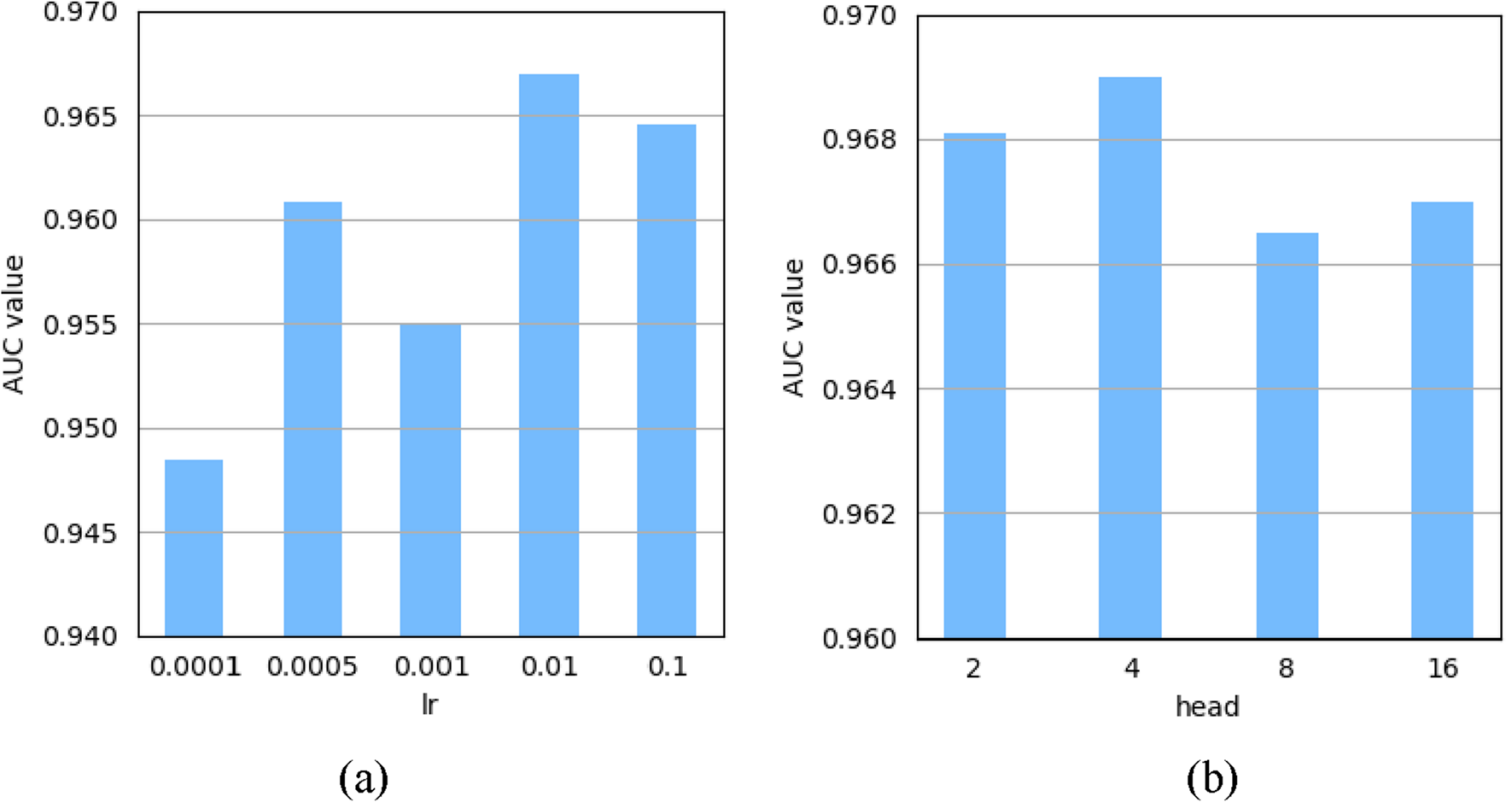
Analysis of the effect of hyperparameters on prediction performance of MDASAE. (**a**) and (**b**) show respectively the AUC values achieved by MDASAE with different learning rates and head numbers in multi-head attention mechanism.

During experiments, we first evaluated the effect of the learning rate *l*_*r*_ on the prediction performance MDASAE while its value varies in the range of {0.0001, 0.0005, 0.001, 0.01, 0.1}. It can be seen from Fig. 2a that MDASAE achieved the highest AUC value while *l*_*r*_ was set to 0.01. After that, we validated the effect of the number of attention mechanism heads *h* in SAE on the prediction performance MDASAE while the value of *h* changes in the range of {2,4,8,16}. From observing the Fig. 2b, it is obvious that the AUC values reached the maximum while* h* was set to 4. Finally, as for the neurons number *n*_1_ of the hidden layer and the number* n*_2_ of stacked AEs in SAE, we found that their values had little effect on the predictive performance of MDASAE through intensive experiments, therefore, we set *n*_1_ and *n*_2_ to their default values {128, 64, 32} and 3 respectively.

### Analysis of the multi-head attention mechanism

**Figure 3:**
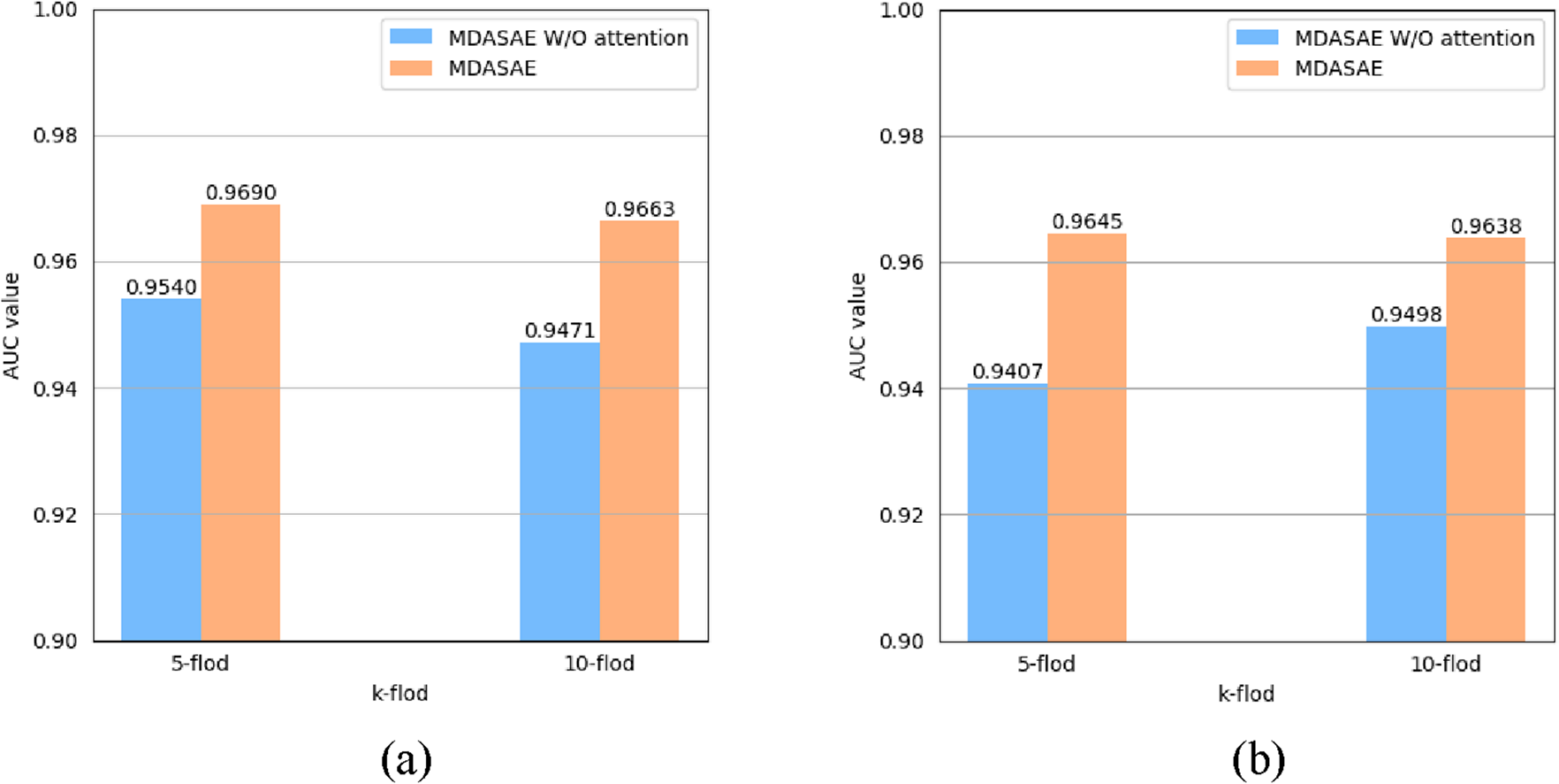
Effect of the multi-head attention mechanism on model performance of MDASAE. (**a**) and (**b**) show the AUC values achieved by MDASAE (with the multi-head attention mechanism) and MDASAE W/O attention (without the multi-head attention mechanism) under the 5-fold cross-validation and the 10-fold cross-validation on MDAD and aBiofilm, respectively.

In MDASAE, we incorporate a multi-head attention mechanism into SAE to help the model jointly focus on information from different representation subspaces at different positions^29^, which will be helpful for the model to capture fruitful feature information. In this section, ablation experiments will be performed based on MDAD and aBiofilm under the 5-fold cross-validation and the 10-fold cross-validation to evaluate the impact of the multi-head attention mechanism on the predictive performance of MDASAE. In the ablation experiment, we will compare the predictive performance between MDASAE (with the multi-head attention mechanism) and MDASAE W/O attention (without the multi-head attention mechanism). As shown in Fig. 3, it is easy to see that MDASAE can achieve higher AUC values when the attention mechanism is introduced.

### Comparison with state-of-the-art predictive methods

In order to further validate the prediction performance of MDASAE, in this section, we will compare it with the following five representative competitive methods based on MDAD and aBiofilm separately:

- LRLSHMDA^30^ adopted the Laplace regularized least squares classifier, a semi-supervised computational model, to predict potential microbe-disease associations.
- HMDAKATZ^12^ proposed a KATZ measure-based calculation method to infer latent associations between microorganisms and drugs.
- BIRWMP^31^ designed a relevance of multi-path based bi-random walk to detect possible microbe-disease associations.
- NTSHMDA^32^ established a random walk algorithm to infer potential microbe-disease associations by integrating network topological similarity.
- LAGCN^33^ combined embeddings from multiple graph convolutional layers with an attention mechanism to predict latent microbe-disease associations.

**Figure 4:**
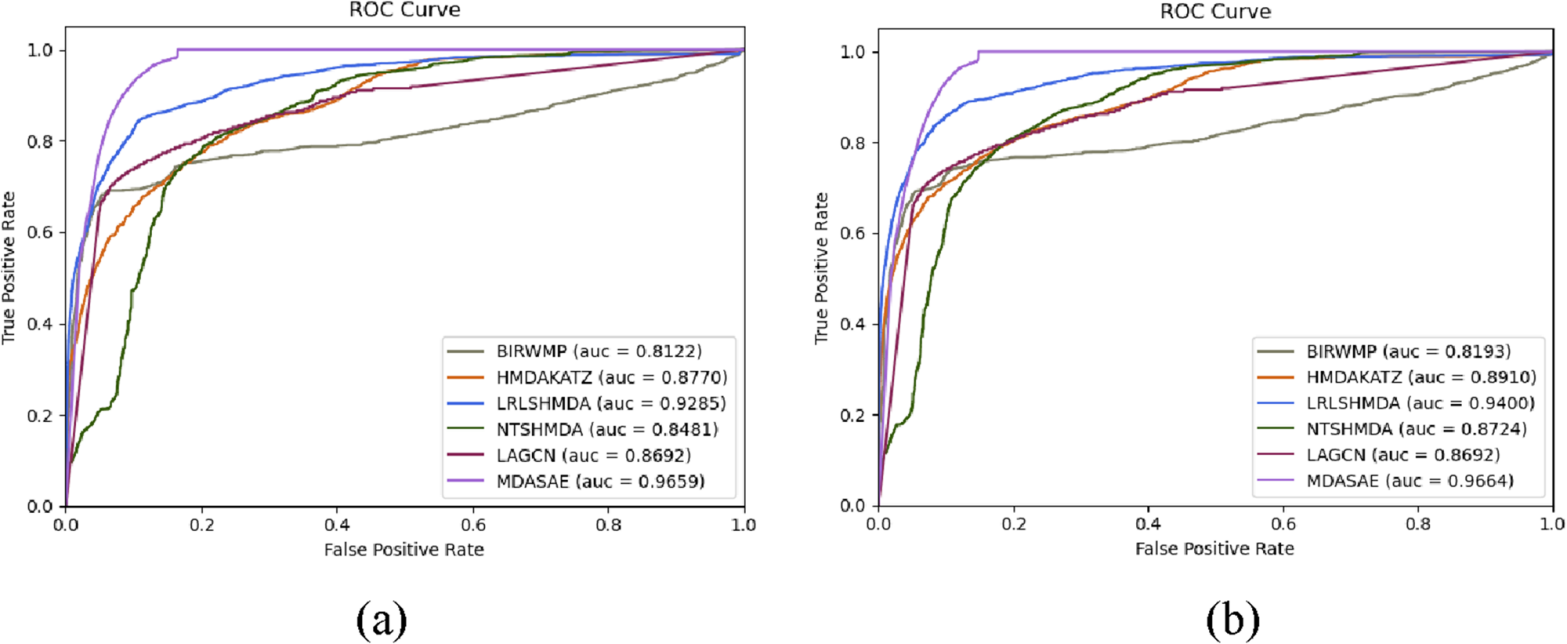
ROC curves achieved by competitive methods based on the MDAD database. (**a**) and (**b**) show the comparison results under 5-fold CV and 10-fold CV respectively.

During experiments, we employed the AUC value as a performance metric while performing 5-fold CV and 10-fold CV for all competitive methods, and illustrated the comparison results in the following Tables 2, 3, and Fig. 4 respectively. Especially, to be fair, all these five competing methods are performed based on their original parameters in comparison experiments.

**Table 2:** Comparison of AUCs achieved by MDASAE and 5 competitive methods under 5-fold CV and 10-fold CV based on MDAD.

Methods	AUC (5-flod)	AUC (10-flod)
LRLSHMDA	0.9259 ± 0.0031	0.9392 ± 0.0014
HMDAKATZ	0.8718 ± 0.0032	0.8928 ± 0.0024
BIRWMP	0.8140 ± 0.0049	0.8172 ± 0.0022
NTSHMDA	0.8495 ± 0.0028	0.8715 ± 0.0020
LAGCN	0.8544 ± 0.0042	0.8637 ± 0.0036
MDASAE (our model)	**0.9665 ± 0.0016**	**0.9659 ± 0.0044**

Significant values are in [bold].

**Table 3:** Comparison of AUCs achieved by MDASAE and 5 competitive methods under 5-fold CV and 10-fold CV based on aBiofilm.

Methods	AUC (5-flod)	AUC (10-flod)
LRLSHMDA	0.9371 ± 0.0023	0.9520 ± 0.0007
HMDAKATZ	0.8988 ± 0.0034	0.9193 ± 0.0014
BIRWMP	0.8491 ± 0.0027	0.8486 ± 0.0026
NTSHMDA	0.8625 ± 0.0023	0.8845 ± 0.0015
LAGCN	0.8615 ± 0.0084	0.8722 ± 0.0074
MDASAE (our model)	**0.9634 ± 0.0007**	**0.9636 ± 0.0003**

Significant values are in [bold].

From observing Table 2, it is easy to see that MDASAE can achieve the highest AUC values of 0.9665 $\pm$ 0.0016 and 0.9659 $\pm$ 0.0044 under the 5-fold CV and 10-fold CV, respectively, followed by LRLSHMDA with AUC values of 0.9259 $\pm$ 0.0031 and 0.9392 $\pm$ 0.0014, whereas BIRWMP with the lowest AUC values.

To further evaluate the predictive performance of MDASAE, we conducted validation on the database of aBiofilm as well. As shown in Table 3, it is obvious that MDASAE can achieve an AUC value of 0.9634 $\pm$ 0.0007 and 0.9636 $\pm$ 0.0003 under 5-fold CV and 10-fold CV, respectively. Similarly, LRLSHMDA ranked second, and BIRWMP was the lowest.

From above descriptions, it is easy to know that MDASAE exhibits the best prediction performance and outperform all these state-of-the-art competing calculation models.

## Case study

To further demonstrate the validity of MDASAE, we will perform case studies of two popular drugs (Pefloxacin and Ciprofloxacin) and a microbe (Mycobacterium tuberculosis) in this section. Among them, Pefloxacin is a fluorinated quinolone that has a broad spectrum of activity against a vast array of Gram-negative and Gram-positive bacteria^34^. In addition, it has been shown that pefloxacin penetrates into cells and is highly effective in the treatment of infections caused by intracellular pathogens^35^, and it is also able to inhibit the activity of a variety of bacteria. For example, El-Sukhon et al. verified the pefloxacin combination was synergistic against *E. coli*^36^, Juvin et al. demonstrated the in vivo bactericidal effect of pefloxacin in an experimental model of endocarditis in Serratia marcescens^37^, and Moin et al. proposed the ability to use pefloxacin as an alternative marker to detect the susceptibility of Salmonella enterica serotypes typhoid and paratyphoid to ciprofloxacin^38^. As shown in Table 4, among the top 20 predicted candidate microbes, there are 16 microbes having been confirmed to be associated with Pefloxacin by previously published literatures.

**Table 4:** The top 20 predict Pefloxacin-associated microbes.

Microbe	Evidence	Microbe	Evidence
*Staphylococcus aureus*	PMID: 2940215	*Vibrio harveyi*	Unconfirmed
*Pseudomonas aeruginosa*	PMID: 1645509	*Bacillus subtilis*	PMID: 12024980
*Escherichia coli*	PMID: 14659660	Human immunodeficiency virus 1	PMID: 9495677
*Candida albicans*	PMID: 11563831	*Actinomyces oris*	Unconfirmed
*Streptococcus mutans*	Unconfirmed	*Streptococcus sanguis*	PMID: 1666667
*Staphylococcus epidermidis*	PMID: 2606159	*Serratia marcescens*	PMID: 8031065
*Staphylococcus epidermis*	PMID: 2640275	*Clostridium perfringens*	PMID: 3162143
*Haemophilus influenzae*	PMID: 2940213	*Streptococcus pneumoniae*	PMID: 20384283
*Salmonella enterica*	PMID: 31954032	*Mycobacterium tuberculosis*	PMID: 1909062
*Enterococcus faecalis*	PMID: 2258345	*Candida glabrata*	Unconfirmed

The first column records top 10 microbes, while the third column records top 11–20 microbes.

In addition, Ciprofloxacin, which is one of the new generations of fluoroquinolone-containing drugs and is a potent and well-tolerated antibacterial drug^39^, has enormous potential for antibacterial activity against both Gram-positive and Gram-negative bacteria, as well as pefloxacin. For example, Rehman et al. mentioned that ciprofloxacin is frequently used for the treatment of various infections caused by the opportunistic pathogen Pseudomonas aeruginosa in their research on the resistance mechanism of ciprofloxacin^40^, Gould investigated the effect of ciprofloxacin to inhibit the activity of pneumococci^41^, and Gollapudi validated the effect of ciprofloxacin to inhibit TNF-$\alpha$ induced HIV expression in U1 cells^42^. As presented in Table 5, among the top 20 predicted candidate microbes, there are 19 microbes having been verified to be associated with Ciprofloxacin by available journals. Thus, it means that MDASAE is helpful for both the clinical application of drugs and prediction of potential drug-associated microbes.

**Table 5:** The top 20 predict Ciprofloxacin-associated microbes.

Microbe	Evidence	Microbe	Evidence
*Escherichia coli*	PMID: 2325984	*Salmonella enterica*	PMID: 32747937
*Pseudomonas aeruginosa*	PMID: 30605076	*Mycobacterium tuberculosis*	PMID: 30020039
*Candida albicans*	PMID: 35404123	*Vibrio harveyi*	PMID: 27247095
*Staphylococcus aureus*	PMID: 35301951	*Stenotrophomonas maltophilia*	PMID: 18510823
*Streptococcus mutans*	PMID: 33402618	Human immunodeficiency virus 1	PMID: 9566552
*Staphylococcus epidermidis*	PMID: 2327776	*Proteus vulgaris*	PMID: 34638966
*Staphylococcus epidermis*	PMID: 10632381	*Serratia marcescens*	PMID: 2071875
*Haemophilus influenzae*	PMID: 29655917	*Actinomyces oris*	Unconfirmed
*Bacillus subtilis*	PMID: 30758259	*Clostridium perfringens*	PMID: 16701569
*Enterococcus faecalis*	PMID: 23789048	*Streptococcus pneumoniae*	PMID: 15155208

The first column records top 10 microbes, while the third column records top 11–20 microbes.

As far as microbes are concerned, Mycobacterium tuberculosis is a category of bacteria that is exclusively aerobic, and it is the primary reason for death due to a single source of infection as the causative agent of human tuberculosis^43^. And the pathogen is universally latent in the human body and can threaten human health any time. Researchers are also searching for various drugs to combat its resistance. For instance, Gaidukevich et al. showed that liposomes of the non-antibiotic levofloxacin containing phospholipid cardiolipin affected the growth of extensively drug resistant Mycobacterium tuberculosis^44^, and Wang et al.^45^ mentioned that ethambutol, as one of the first-line antituberculosis drugs, has a resistance rate of 17.2% against multi-drug resistant tuberculosis. In Table 6, all these top 20 Mycobacterium tuberculosis-associated candidate drugs predicted by MDASAE have been confirmed by published reports.

**Table 6:** The top 20 predict mycobacterium tuberculosis-associated drugs.

Drug	Evidence	Drug	Evidence
Ciprofloxacin	PMID: 30020039	Capreomycin	PMID: 21678479
Tobramycin	PMID: 19723387	Viomycin	PMID: 14799786
Pyrazinamide	PMID: 34181476	Triclosan	PMID: 19130456
Epigallocatechin Gallate	PMID: 33463343	SQ109	PMID: 22258923
Limonene	PMID: 29288759	Ceftazidime	PMID: 32773662
Bedaquiline	PMID: 33055230	Rifapentine	PMID: 33856282
Isoniazid	PMID: 33132303	Levofloxacin	PMID: 30029913
Aminosalicylic Acid	PMID: 26033719	Curcumin	PMID: 23305394
Verapamil	PMID: 30648892	Ethambutol	PMID: 27806932
Colistin	PMID: 26183185	Oxacillin	PMID: 33109697

The first column records top 10 drugs, while the third column records top 11–20 drugs.

From above results of case studies, it is easy to see that MDASAE may be a promising tool for predicting potential associations between microbes and drugs in the future.

## Discussion and conclusion

Researchers have shown that there is a mutually constraining and interdependent relationship between humans and microbes, and a substantial portion of microbes are closely related to human health. Normally, the probability of infectious diseases in the human body is minimal, and the microbes that reside in the human body are harmless, and they are even resistant to pathogens, thus, it also promotes research on the prediction of the association between microbes and drugs.

In this work, we proposed a novel prediction model MDASAE for inferring latent microbe-drug associations. In MDASAE, we first constructed different microbe similarity networks and drug similarity networks based on known linkage data information. And then, some of them were used as input to SAE to learn attribute features for microbes and drugs, and the remaining of them were combined with these newly-learned attribute features to predict scores of possible associations between microbes and drugs. Results of case study and intensive comparison experiments showed that MDASAE was superior to existing competitive state-of-the-art calculation methods, which indicated that MDASAE might be a promising tool for identifying latent microbe-drug associations, and had potential for new drug discovery and drug clinical treatment at the same time. Furthermore, we may be able to apply MDASAE to other association prediction issues between biological entities, such as microbe-disease association prediction and circRNA-disease association prediction, etc.

Although MDASAE has some advantages, there will still be some limitations as well. For instance, some of these microbes predicted to be related to specific drugs by MDASAE showed less evidence of association with each other. And additionally, SAE cannot stack too many AEs owing to the sparsity of the dataset, which tends to cause overfitting phenomena. In the future, we will consider data augmentation to solve this problem (“Supplementary information”).

## Supplementary Information

Supplementary Information 1.

Supplementary Information 2.

Supplementary Information 3.

Supplementary Information 4.

Supplementary Information 5.

Supplementary Information 6.

Supplementary Information 7.

Supplementary Information 8.

## Data Availability

The original contributions presented in the study are included in the article, further inquiries can be directed to the corresponding author.
